# Diagnostic Accuracy of MALDI-TOF Mass Spectrometry for the Direct Identification of Clinical Pathogens from Urine

**DOI:** 10.1515/med-2020-0038

**Published:** 2020-04-04

**Authors:** Min Tang, Jia Yang, Ying Li, Luhua Zhang, Ying Peng, Wenbi Chen, Jinbo Liu

**Affiliations:** 1 Department of Laboratory Medicine, Affiliated Hospital of Southwest Medical University, Luzhou City, Sichuan Province, China; 2 Department of Pathogenic Biology, College of Preclinical Medicine, Southwest Medical University, Luzhou city, Sichuan Province, China

**Keywords:** MALDI-TOF MS, Direct identification, Urinary tract infection, Urine culture, Diagnostic accuracy

## Abstract

Matrix-assisted laser desorption ionization time of flight mass spectrometry (MALDI-TOF MS) has become one of the most popular methods for the rapid and cost-effective detection of clinical pathogenic microorganisms. This study aimed to evaluate and compare the diagnostic performance of MALDI-TOF MS with that of conventional approaches for the direct identification of pathogens from urine samples. A systematic review was conducted based on a literature search of relevant databases. The pooled sensitivity, specificity, positive likelihood ratio (PLR), negative likelihood ratio (NLR) and area under the summary receiver operating characteristic (SROC) curve of the combined studies were estimated. Nine studies with a total of 3920 subjects were considered eligible and included in the meta-analysis. The pooled sensitivity was 0.85 (95% CI 0.79-0.90), and the pooled specificity was 0.93 (95% CI 0.82-0.97). The PLR and NLR were 11.51 (95% CI 4.53-29.26) and 0.16 (95% CI 0.11-0.24), respectively. The area under the SROC curve was 0.93 (95% CI 0.91-0.95). Sensitivity analysis showed that the results of this meta-analysis were stable. MALDI-TOF MS could directly identify microorganisms from urine samples with high sensitivity and specificity.

## 1 Introduction

Urinary tract infections (UTIs) are among the most common clinical infectious diseases and are also a major cause of hospital-acquired infections [1,2]. The clinical symptoms of UTIs range from simple cases such as cystitis to severe cases such as uroseptic shock. In addition, the etiology of UTIs varies, although *Escherichia coli* is the leading causative agent, and pathogen resistance to common antibiotics largely depends on the geographical location [3]. Currently, the definitive diagnosis of urinary tract infections relies on urine culture [4]. Typical laboratory diagnosis of a UTI requires culture of the pathogen for 18-48 hours, and antibiotic susceptibility testing results require an additional 24-48 hours [5]. Before a final diagnosis is obtained, the UTI patient may be treated empirically with an inappropriate antimicrobial therapy, which could lead to a higher mortality rate [6]. Hence, the rapid and correct identification of pathogens from urine samples is urgent and important.

In recent years, protein analysis based on the technique matrix-assisted laser desorption ionization time of flight mass spectrometry (MALDI-TOF MS) has been implemented and is known as a rapid and reliable method for bacterial identification [7]. Several institutions have applied this method to identify bacteria in conjunction with antibiotic stewardship, which aids in providing timely antibiotic therapy and decreasing unnecessary antibiotic use [8, 9, 10]. In light of its promise, MALDI-TOF MS technology was described as “a revolution in clinical microbiology” [11] . Currently, four main commercial systems are in more popular use worldwide: the MALDI Biotyper (Bruker Daltonics, Germany), the Vitek MS (bioMérieux, France) ,Shimadzu and Applied Biosystems [12,13]. Each system has its own characteristics. Many studies have reported the direct detection and identification of bacterial pathogens from urine samples using MALDI-TOF MS [14, 15]. However, these studies only included a few strains, and the results are somewhat inconsistent.

Therefore, the present work aimed to analyze and compare the performance of MALDI-TOF MS with that of common methods for the diagnosis of pathogens from urine samples by performing a meta-analysis that synthesized large amounts of data to improve the reliability of the results.

## 2 Materials and methods

### 2.1 Search strategy

We systematically searched original papers published in PubMed, Embase_，_Web of Science, and the China National Knowledge Infrastructure (CNKI) databases. These databases were queried with the following keywords and subject terms: “maldi” or “Matrix-assisted Laser Desorption Ionization”, “urine” or “urinary tract infections” or “infection”, and “identification”. Disagreements were resolved by consultation with a third researcher. To obtain relevant studies, the articles were first screened by title and abstract; then, full articles were further evaluated. The search was limited to publications in Chinese or English. We searched the databases for relevant articles published from inception to March 31, 2018.

### 2.2 Inclusion and exclusion criteria

Studies evaluating and comparing the accuracy of MALDI-TOF MS with that of routine identification methods for the identification of clinical pathogens in urinary tract infections were considered eligible for the meta-analysis. Typically, routine identification methods included the Vitek II system, API system, MicroScan WalkAway system or other routine biochemical tests and/or 16S rRNA sequencing (molecular biology). We required sufficient information to construct 2Í2 contingency tables. We contacted the authors for additional data for analysis if needed.

We also applied the following exclusion criteria: (i) studies that did not investigate urinary tract infections; (ii) studies lacking urine specimens; (iii) studies lacking reference methods, a comparator method or gold standard; and (iv) studies identifying clinical pathogens by mass spectrometry methods other than MALDI-TOF MS.

### 2.3 Data extraction

Two reviewers (MT and JY) independently extracted pertinent data from each study. Any inconsistencies were resolved in a consensus meeting or through discussion with the third author (YL). The quality of eligible studies was assessed by using the Quality Assessment of Diagnostic Accuracy Studies (QUADAS) 2 questionnaire [16]. The following data were included: first author, year of publication, country, number of patients, MALDI-TOF MS system, TP (true positive), FP (false positive), FN (false negative), and TN (true negative), and the method of specimen handling.

### 2.4 Statistical analysis

We adopted the recommended standard methods for a meta-analysis of diagnostic research evaluations [17]. Analyses were performed using STATA version 12.0. To obtain accurate and objective results, we defined the following terms used to assess the sensitivity and specificity of MALDI-TOF MS identification: true positive: the MALDI-TOF MS and culture results were consistent, and only one microorganism was isolated in culture; false positive: the MALDI-TOF MS and culture results differed; false negative: a positive culture in the absence of MALDI-TOF MS identification; and true negative: no MALDI-TOF MS identification of samples with a negative culture.

The summary receiver operating characteristic (SROC) curve was plotted to depict a simultaneous non-linear relationship between sensitivity and specificity and calculated to evaluate the overall diagnostic performance of MALDI-TOF MS. Statistical heterogeneity was tested through the Q statistic and the inconsistency index (I^2^) (a P value of Q<0.05 or I^2^ >50% was considered the threshold indication of heterogeneity). The source of heterogeneity was further investigated by using sensitivity analysis that ascertained whether the results were stable.

Publication bias was determined by performing Deek’s funnel plot asymmetry test. Statistical significance was defined as *P* < 0.05 or an asymmetric funnel plot.

## 3 Results

### 3.1 Results of the systematic literature search

**Figure 1:**
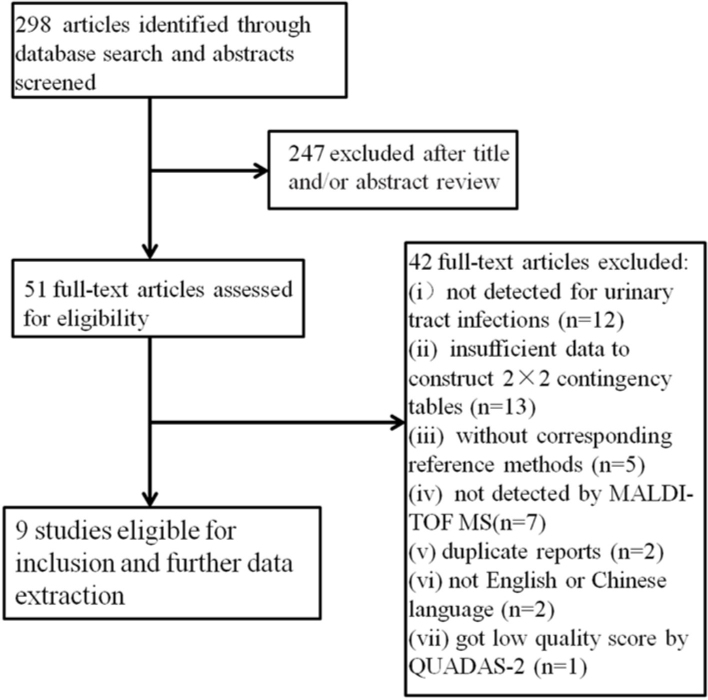
Flowchart describing the systematic literature search and study selection process for the meta-analysis.

A total of 298 studies were retrieved by searching the indicated databases. After reviewing titles and/or abstracts, we excluded 247 articles. We further excluded 42 studies based on the inclusion and exclusion criteria. Finally, 9 studies were considered eligible and used in the meta-analysis (Figure 1).

### 3.2 Study characteristics

The main characteristics of the enrolled eligible studies are shown in Table 1. These studies originated from 5 countries and had a total of 3920 subjects. The most common reference method for pathogen identification was a biochemistry test after incubating for 18-48 hours. Among the included articles, six studies [18, 19, 20, 21, 22, 23] reported on the identification performance of the Biotyper system, and the remaining three studies [24, 25, 26] reported on the identification performance of the Vitek MS system.

**Table 1:** Main characteristics of the enrolled studies

Author	Year	Country	N_1_^^a^^	N_2_^^b^^	N	TP	FP	FN	TN	system	specimen handling^^c^^
Ferreira (18)	2010	Spain	260	5	255	205	2	28	20	MALDI Biotyper	ICM and PEM
Ferreira (19)	2011	Spain	238	0	238	193	11	14	20	MALDI Biotyper	ICM and PEM
Wang (20)	2013	China	1456	44	1412	387	8	35	982	MALDI Biotyper	PEM
Burillo (21)	2014	Spain	207	8	199	130	8	36	25	MALDI Biotyper	PEM
Veron (24)	2015	France	103	6	97	74	1	11	11	VITEK MS	Short time culture
Haiko (25)	2016	Finland	207	49	158	94	2	46	16	VITEK MS	Short time culture
Zboromyrska (22)	2016	Spain	140	36	104	89	0	12	3	MALDI Biotyper	PEM
Huang (26)	2017	China	1167	9	1158	295	69	47	747	VITEK MS	PEM
Kitagawa (23)	2017	Japan	142	0	142	90	0	43	9	MALDI Biotyper	PEM

N: the number of calculated indexes after removing contaminated or/and 2 morphology samples.

a the total number of paired culture samples compared to MALDI-TOF MS

b the number of contaminated or/and 2 morphology colony types

c ICM, intact cell method; PEM, protein extraction method

### 3.3 The methods of specimen handling

In order to obtain a certain amount of bacteria, the urine samples were often concentrated. The enrolled studies included roughly three methods for specimen handing prior to MALDI-TOF MS measurement (Table 1). Ferreira L et.al [19] described the general workflow of ICM （intact cell method）and PEM（protein extraction method).

### 3.4 Overall meta-analysis

**Figure 2:**
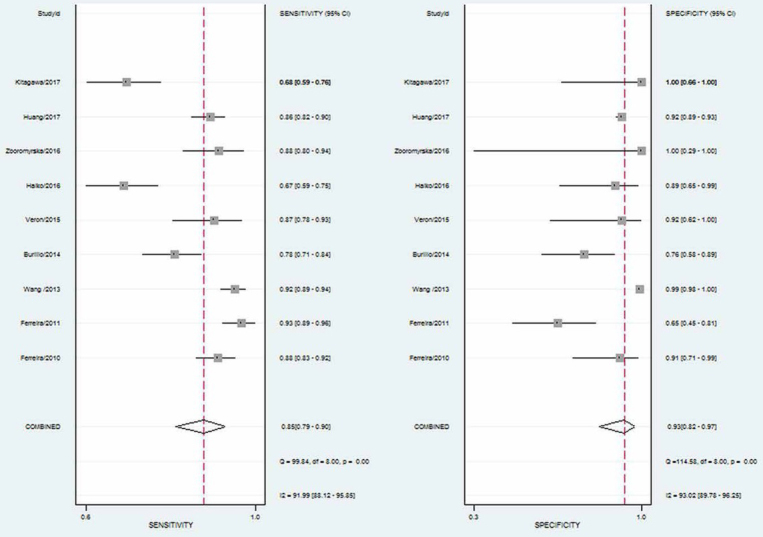
Forest plot of the pooled sensitivity and specificity of MALDI-TOF MS for identifying pathogens.

**Figure 3:**
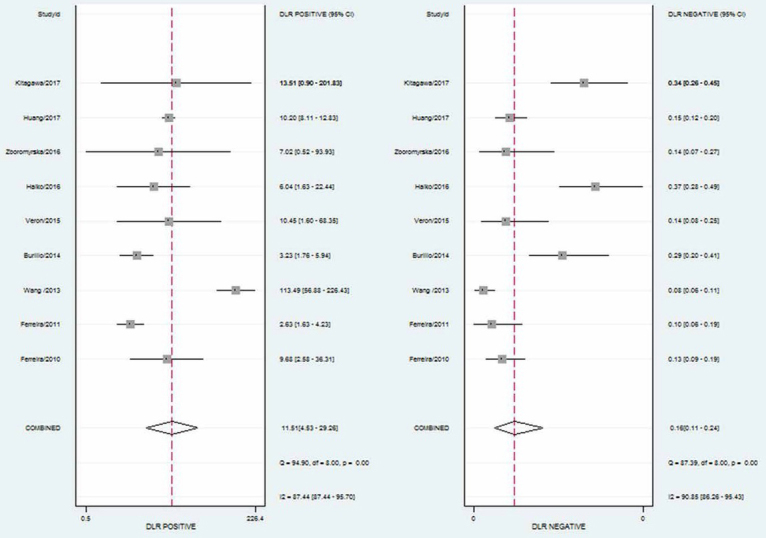
Forest plot of positive likelihood ratio and negative likelihood ratio.

**Figure 4:**
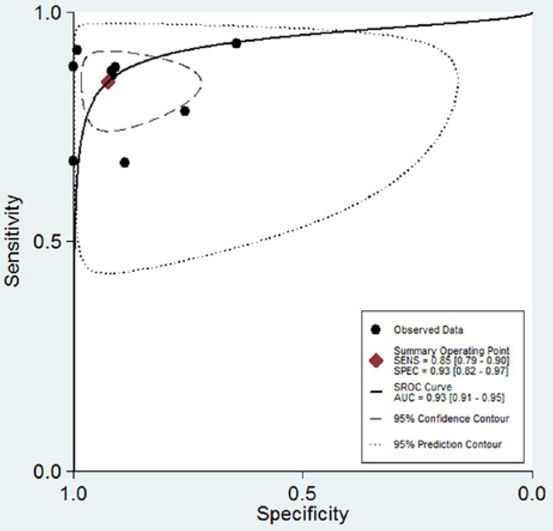
Summary receiver operating characteristic curve. The figure also shows 95% confidence contour and 95%prediction contour.

Notably, current MALDI-TOF MS data software analysis is unable to reliably identify all microorganisms present in a mixture of microorganisms [27,28]. As such, contaminated urine samples often appear to produce insignificant results. Therefore, we calculated indexes after removing such samples. The pooled sensitivity was 0.85 (95% CI 0.79-0.90), and the pooled specificity was 0.93 (95% CI 0.82-0.97; Figure 2). The PLR and NLR were 11.51 (95% CI 4.53-29.26) and 0.16 (95% CI 0.11-0.24), respectively (Figure 3). The area under the receiver operating characteristic curve (SROC) was 0.93 (95% CI 0.91-0.95; Figure 4).

### 3.5 Performance of MS systems

The meta-analysis included two MS systems: the MALDI Biotyper and the Vitek MS. We compared the diagnostic accuracy of the MALDI Biotyper and the Vitek MS and found that the estimated specificity and sensitivity of all studies using the MALDI Biotyper were 0.94 (95% CI 0.75-0.99) and 0.86 (95% CI 0.79-0.91), respectively, while the specificity and sensitivity of the Vitek MS were 0.91 (95% CI 0.87-0.94) and 0.81 (95% CI 0.70-0.89), respectively.

### 3.6 Heterogeneity analysis and sensitivity analysis

There was substantial heterogeneity among the studies (overall *I*^2^for the bivariate model: 95%, 95% CI 91-99 and *P*<0.001). However, we recorded no evidence of a threshold effect. Therefore, we further investigated the source of heterogeneity with a sensitivity analysis. This analysis was performed by inspecting pooled estimates that were calculated by omitting one study at a time. As shown in Supplementary Figure S1, there was no evidence that any individual study had an obvious effect on the combined overall results. Hence, the results of this meta-analysis were stable.

### 3.7 Assessment of publication bias

Publication bias among the included studies was analyzed by performing Deek’s funnel plot asymmetry test using STATA software. The results indicated that there was no publication bias among studies (*P*=0.15) (Supplementary Figure S2).

## 4 Discussion

As one of the most popular technologies in clinical microbiology, MALDI-TOF MS demonstrates great advantages for microbial species identification [29]. Currently, MALDI-TOF MS is considered the holy grail of rapid and cheap microbial identification. Moreover, it produces final results more quickly than conventional methods, and it directly identifies microbes from positive blood cultures, which may enhance the quality of patient management [30]. In this meta-analysis, we performed a systematic review and evaluated the ability of MALDI-TOF MS systems to accurately and directly identify clinical pathogens in urine samples.

MALDI-TOF MS demonstrated high accuracy for the direct identification of pathogens from urine samples, with a pooled sensitivity of 0.85 and a pooled specificity of 0.93. As reported by Kim et al., higher PLR values indicate the greater likelihood of an association between a test result and a disease, and lower NLR values forecast the greater likelihood that a test result is related to the absence of a disease[31] .Therefore, based on the PLR and NLR values obtained for MALDI-TOF MS, this method is reliable for the direct detection of pathogenic microorganisms from urine samples. Furthermore, the SROC value was 0.93, which indicated a high degree of overall diagnostic accuracy.

This study also investigated the performance of different MS systems. The Biotyper system demonstrated better performance in six studies that were enrolled and synthesized in this meta-analysis, with a higher specificity and sensitivity than that of the Vitek MS system. However, it remains uncertain whether the identification accuracy of the Biotyper system is generally superior to that of the other three systems of interest. On the one hand, the present study only included two different detection systems, and no included study performed a sufficient direct comparison of these systems. On the other hand, identification performance can be influenced by many factors (for example, the reference library version, the number of studies, or the method for handling samples), as revealed by the substantial heterogeneity of the included studies. Therefore, additional studies should be conducted to confirm our results.

To obtain good results with MALDI-TOF MS directly from clinical samples, the bacterial count and pathogenic species seem to be the critical issues. Wang et. al considered that Gram-positive cocci samples with bacterial counts of <105 CFU/ml did not provide reliable MALDI-TOF results and Gram-negative bacilli samples could be accurately identified when bacterial counts were <105 CFU/ml [20]. It migh be possible that the cell wall of Gram-negative bacilli were more easily lysed and the proteins were more easily detected. Therefore, it is necessary to improve the sensitivity of MALDI-TOF MS and reduce the threshold of detection. And to obtain sufficient spectra for MALDI-TOF MS detecting, the sample must be concentrated. The included studies used various concentration procedures to facilitate identification, including a centrifugation (low and high speed)/wash protocol that yields pure microbial pellets and added formic acid and acetonitrile to destroy the cell wall more fully[18, 19, 20, 21, 22, 23, 26]. Ferreira et.al [19] compared the ICM with PEM and showed that the intact cell method (ICM) provided excellent results for urine. They considered that the ICM should initially be applied to all samples and, if reliable identification is not achieved, the PEM should be applied to increase the possibility of identification. An alternative method for concentration was “short incubation”, in which samples are cultured for 3-5 hours followed by sample preparation [24, 25]. Although the “short culture” method required a longer time than direct methods to obtain results, the overall efficiency was similar among all methods. Compared with the conventional method (culture for 18-48 h), these alternative protocols saved a substantial amount of time, which considerably improved patient treatment.

It was not feasible to apply more elaborate meta-regression to investigate the source of heterogeneity with only nine enrolled studies. Therefore, we performed a sensitivity analysis to analyze the heterogeneity. As shown in Supplementary figure S1, the pooled index was not appreciably affected by omitting any single study, which indicated the stability of our results.

We must acknowledge certain limitations of the present work. First, although we attempted to search for all relevant studies, some data were inevitably missing. The studies enrolled in the meta-analysis were restricted only to articles published in Chinese or English. Hence, the number of included studies was small. Second, given the current limitations of the technique, MALDI-TOF MS was unable to identify all microorganisms present in a mixture of microorganisms. Additionally, it was unable to distinguish the most abundant pathogen in a mixture. Therefore, we eliminated samples that were contaminated or contained two or more pathogens, which may have resulted in the overestimation of accuracy. Improved algorithms are needed to interpret the spectra obtained for combinations of bacteria resulting from the direct testing of urine samples [32]. Third, in its current iteration, MALDI-TOF MS does not produce quantifiable results. It is uncertain whether MALDI-TOF MS meets the requirements for a UTI diagnostic method. Clinically, we suggested that urine samples should first be screened by flow cytometry or Gram-staining, and then bacteriuria-positive samples could be directly analyzed with MALDI-TOF MS. Meanwhile, we could not but acknowledge MALDI-TOF MS currently has some other limitations. The cost of purchasing and maintaining the instrument is high, it is difficult to discriminate some closely related species such as *Escherichia coli* and *Shigella* and it is difficult for the method to detect antimicrobial resistance.

The results of the meta-analysis suggested that MALDI-TOF MS could directly identify microorganisms from urine samples with higher sensitivity and specificity than routine methods. More studies should be conducted to confirm the performance this technology and many aspects of MALDI-TOF MS must be improved, such as the ability to provide information about the antimicrobial susceptibility of pathogens in clinical samples.
