# Supplementary material for: Diagnostic Accuracy of MALDI-TOF Mass Spectrometry for the Direct Identification of Clinical Pathogens from Urine
Source: Open Med (Wars). 2020 Apr 4;15:266–73. doi: 10.1515/med-2020-0038 (PMC7147288; doi:10.1515/med-2020-0038)
Supplement: Supplementary file 1 [file med-15-266_sm.pdf]

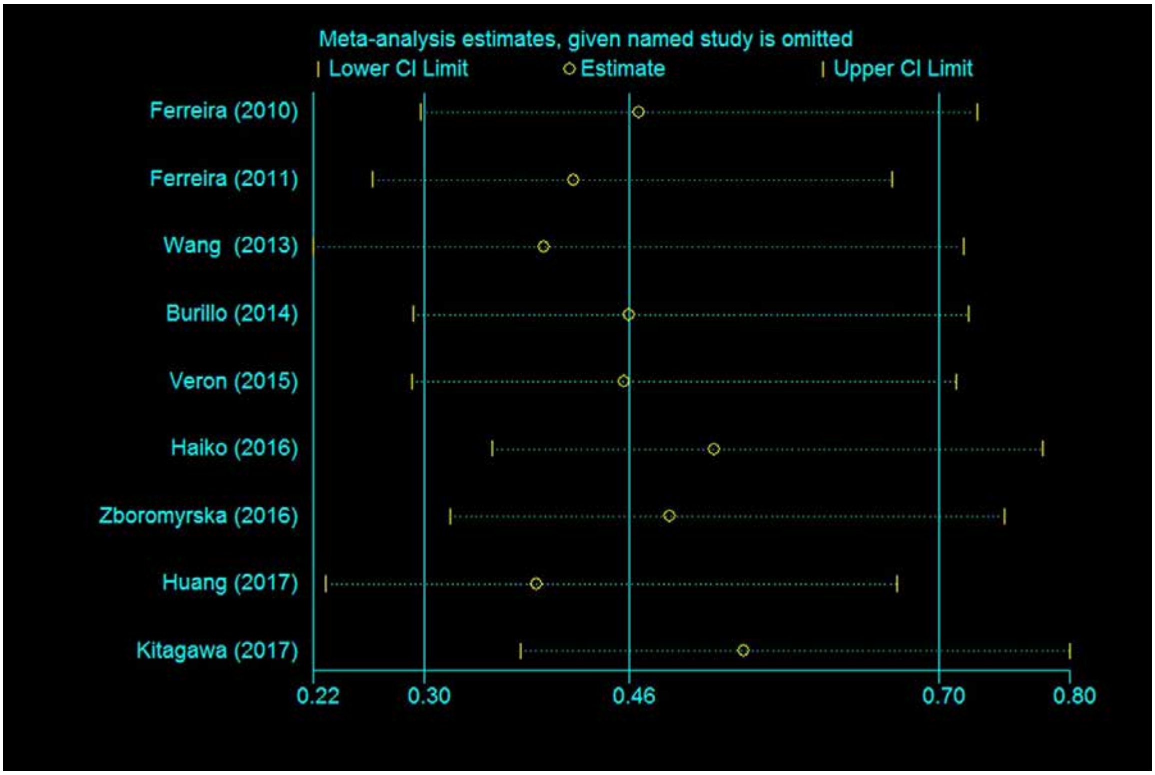

Supplementary figure S1: Sensitivity analysis.

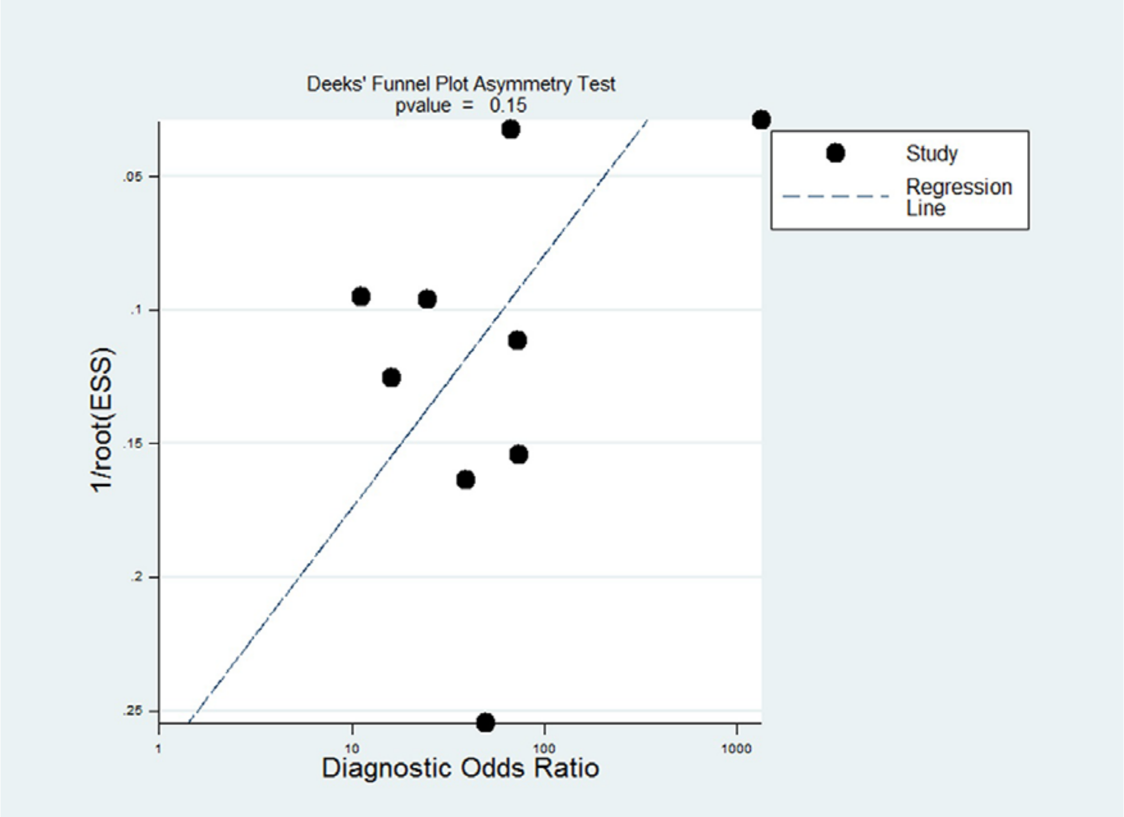

Supplementary figure S2: Deek's funnel plot assessment of potential publication bias.
